# Supplementary material for: Diindoles produced from commensal microbiota metabolites function as endogenous CAR/Nr1i3 ligands
Source: Nat Commun. 2024 Mar 22;15:2563. doi: 10.1038/s41467-024-46559-3 (PMC10960024; doi:10.1038/s41467-024-46559-3)
Supplement: Supplementary file 1 — Supplementary Information [file 41467_2024_46559_MOESM1_ESM.pdf]

Diindoles produced from commensal microbiota metabolites function as endogenous CAR/Nr1i3 ligands

Supplementary Fig. 1 | CAR-LBD-SRC1 interacts well with known CAR ligands.

Supplementary Fig. 2 | Structural annotation of *m/z* 130.0652 by LC-MS.

Supplementary Fig. 3 | Comparison of retention times for purified and pulldown diindoles.

Supplementary Fig. 4 | Melt curves of CAR LBD-SRC1 incubated with 0.4-50 μM of DIM, DIE, DIP, nDIB, and iDIB.

Supplementary Fig. 5 | Effects of CAR ligands on PGC1α recruitment.

Supplementary Fig. 6 | Effects of ligands on transcriptional activity.

Supplementary Fig. 7 | WT female mice treated with diindoles and CAR inverse agonist And.

Supplementary Fig. 8 | Liver-to-body weight ratio in the control and treatment group of both sexes.

Supplementary Fig. 9 | Evaluation of DIE producing ability across a 27-strain Lactobacilli collection.

Supplementary Fig. 10 | Eleluation of diindole levels in the tissues of WT and Nr1i3$^{-/-}$ male mice treated with diindoles.

Supplementary Table 1 | ^{1}H and ^{13}C NMR data of GM-1, GM-2, and GM-7 in methanol-d_4 (δ in ppm, J in Hz).

Supplementary Table 2 | HDX summary table.

Supplementary Table 3 | Primers for gene expression analysis.

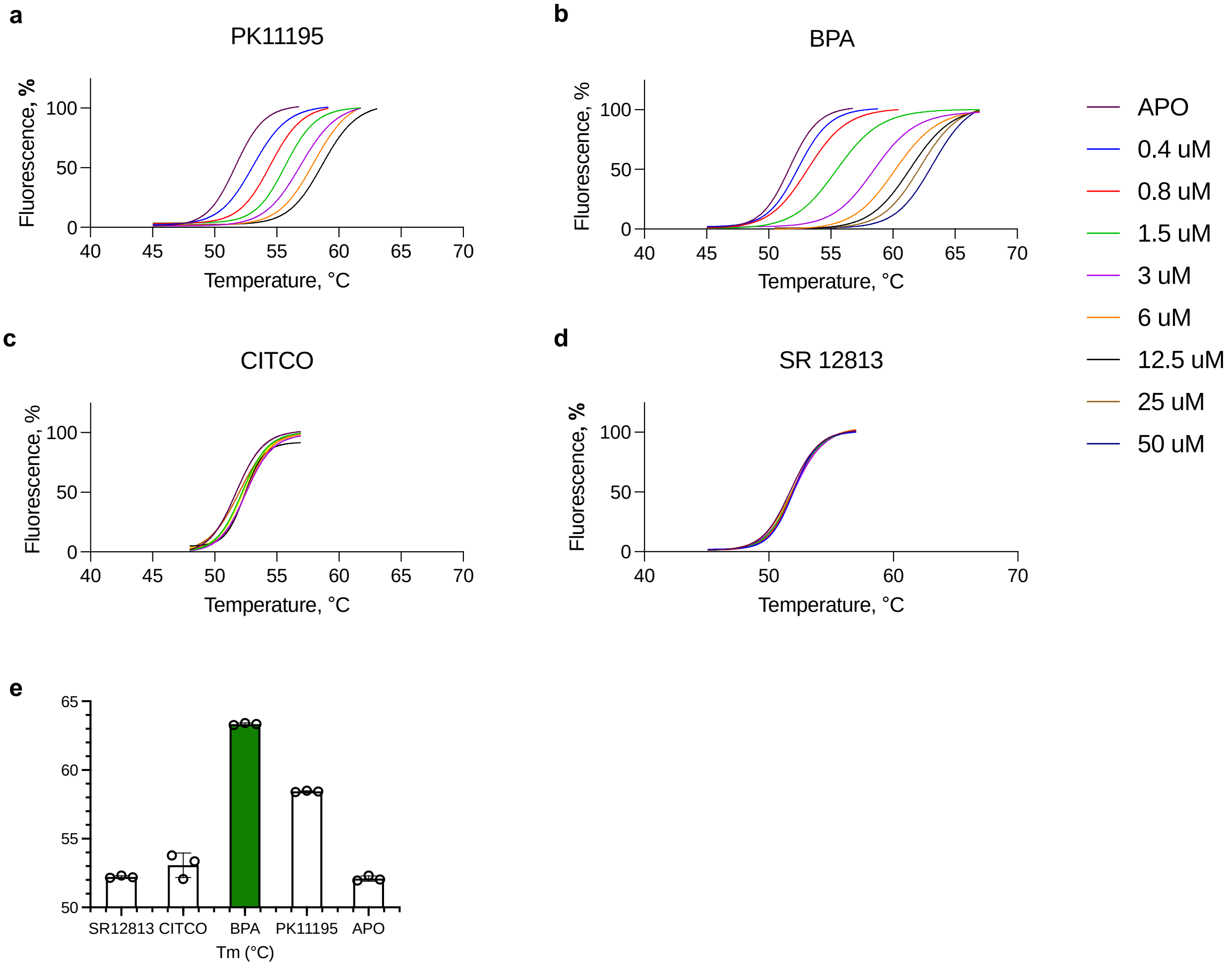

Supplementary Fig. 1 | CAR-LBD-SRC1 interacts well with known CAR ligands.

a–**d**, Thermal shift and dose-response assays demonstrating preferential stabilization of the CAR-LBD-SRC1 fusion by known synthetic CAR ligands **a,** PK11195 and **b,** BPA, but not by **c,** CITCO or **d,** the PXR ligand SR12813. **e,** Maximum thermal shifts. Data were obtained from triplicate experiments and are expressed as means ± SEM.

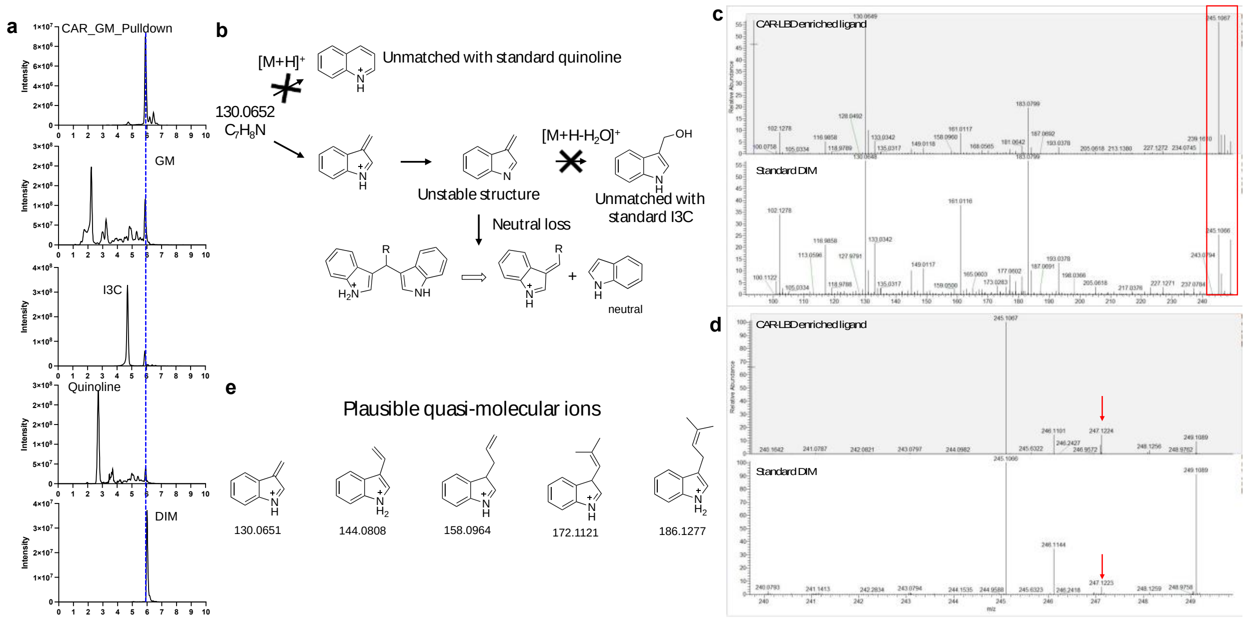

Supplementary Fig. 2 | Structural annotation of *m/z* 130.0652 by LC-MS.

a, Top panel shows the *m/z* 130.0652 chromatogram of the hCAR pulldown sample with the dashed blue line indicating the peak that corresponds with the mass feature in the gut microbiome sample (panel below). The bottom three panels show *m/z* 130.0652 chromatograms of indicated reference standards. **b**, Predicted structure of *m/z* 130.0652. **c**,**d**, MS1 spectra of *m/z* 130.0652 in the hCAR pulldown sample and of the standard DIM. **c**, Full spectra ranging from *m/z* 90 to 250. **d**, Close-up views of the red box area in the top two panels. **e**, Plausible quasi-molecular ions that correspond with each of the mass features of interest.

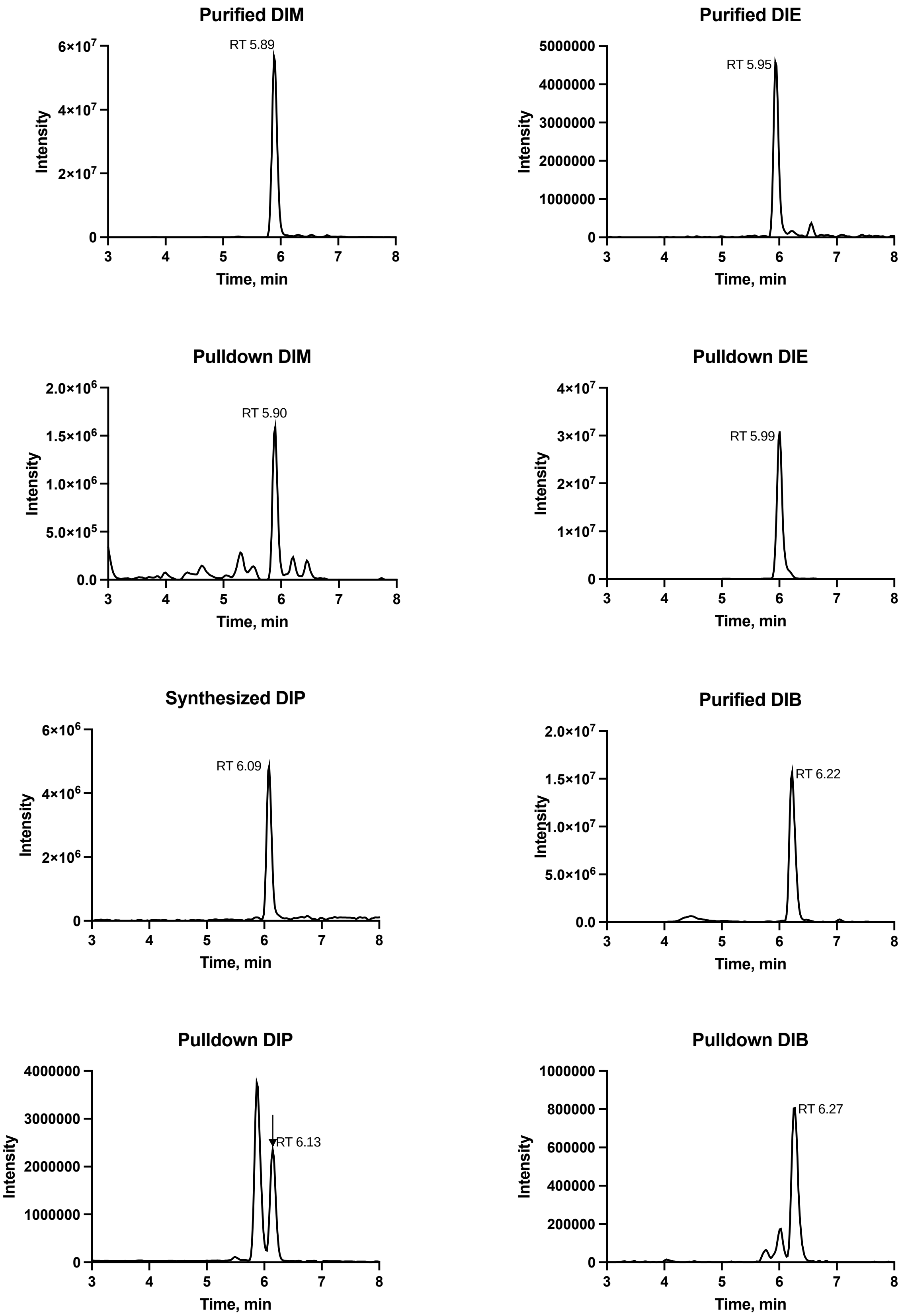

Supplementary Fig. 3 | Comparison of retention times for purified and pulldown diindoles.

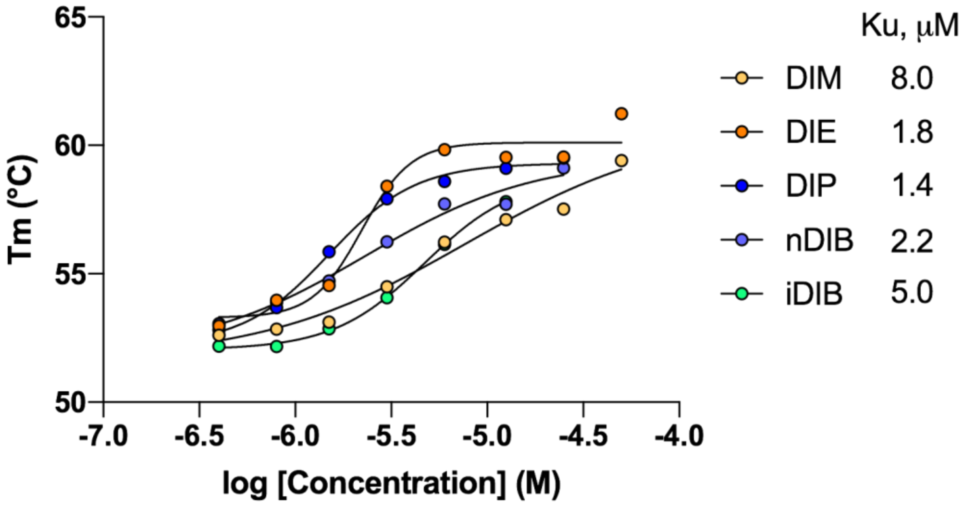

Supplementary Fig. 4 | Melt curves of CAR LBD-SRC1 incubated with 0.4–50 *μ*M DIM, DIE, DIP, nDIB or iDIB. Dose responsive shifts in Tm are shown. The equilibrium between folded and melted protein is reversible and described with constant K_{unfold} (K_{u}).

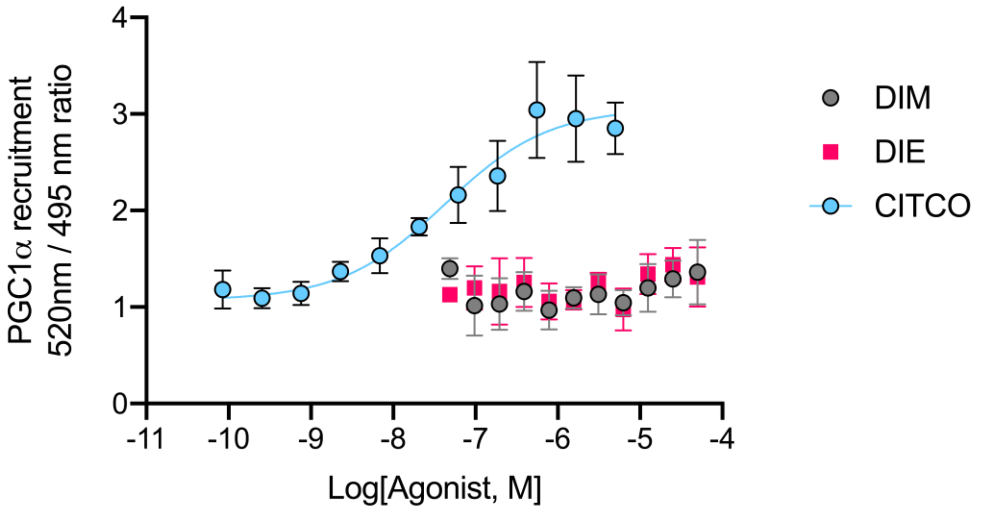

Supplementary Fig. 5 | Effects of CAR ligands on PGC1α recruitment. TR-FRET shows no effects by DIM or DIE on PGC1α peptide recruitment. Results were obtained from experiments performed in quadruplicate. Data are expressed as means ± SD

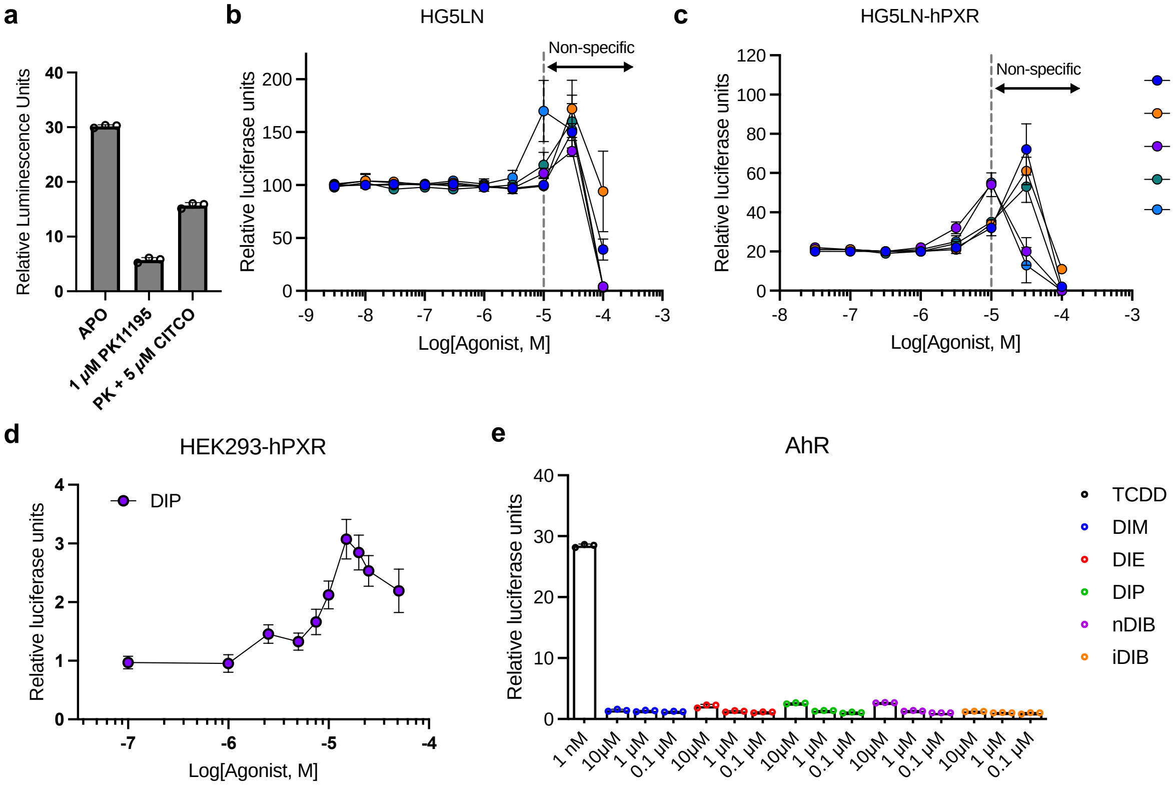

Supplementary Fig. 6 | Effects of ligands on transcriptional activity. a, PK11195 suppresses hCAR luciferase activity and co-treatment with CITCO restores hCAR luciferase activity in HEK293 cells. **b,** Assessing non-specific effects of diindoles on luciferase reporter. HG5LN cells expressing (GAL4RE)5-Luc were treated with increasing amounts of diindoles to evaluate non-specific luminescence in the absence of CAR. Dashed line represents the concentration at which non-specific luminescence is observed (10 μM). **c,** Diindoles except for DIP do not activate GAL4-hPXR in HG5LN cells. **d,** DIP activates GAL4-hPXR at concentrations of 5–15 μM in HEK293 cells. Rifampicin is an authentic hPXR agonist. **e,** Diindoles do not activate GAL4-hAhR in H4IIE cells. 2,3,7,8-Tetrachlorodibenzodioxin (TCDD) is a prototypical hAhR agonist. Results were obtained from experiments performed in triplicate (n = 3 independent experiments). Data are expressed as means ± SD.

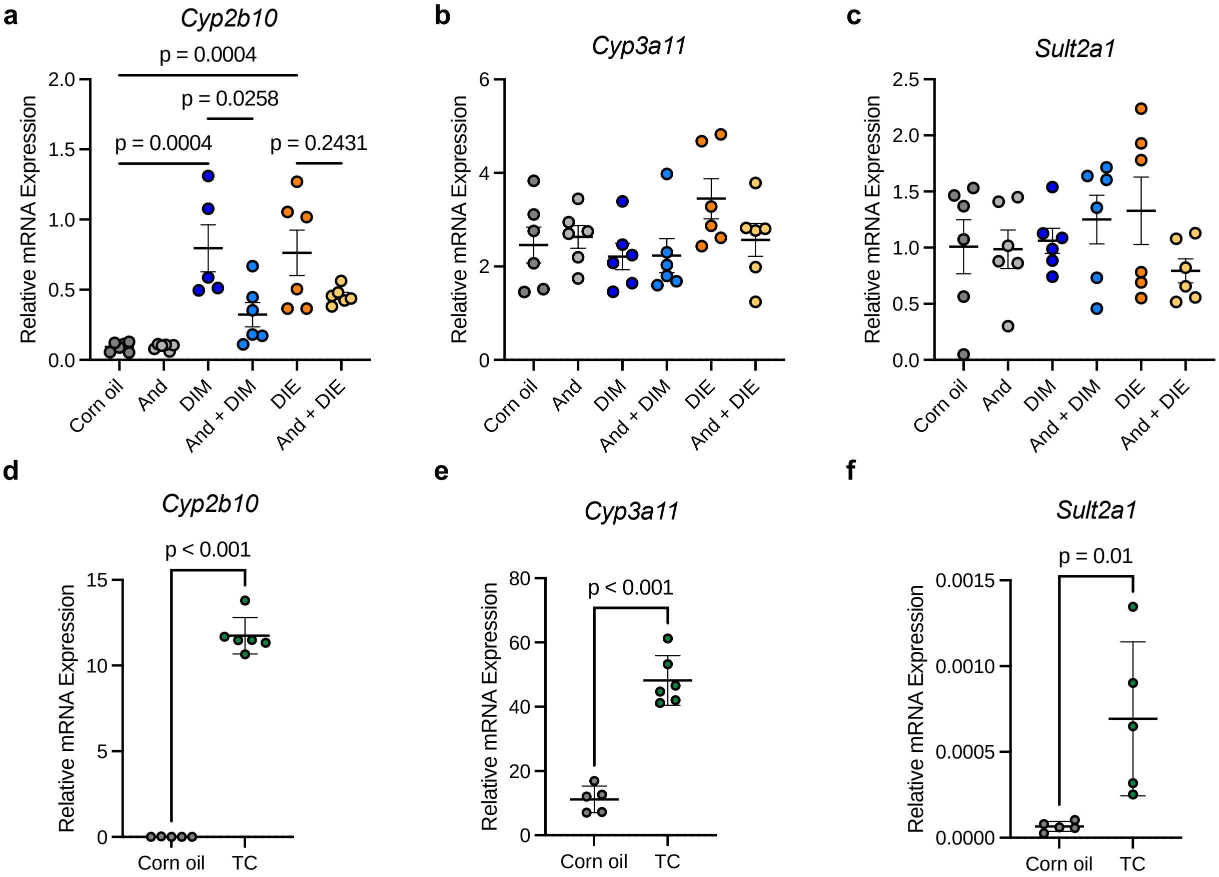

Supplementary Fig. 7 | a–c, WT female mice treated with diindoles and CAR inverse agonist And. d–f, WT male mice treated with TCPOBOP (TC). qPCR analysis of genes **a**,**d,** *cyp2b10*, **b**,**e,** *cyp3a11*, and **c**,**f,** *sult2a1*. (**a**–**c**, $n = 6$, **d**–**f**, $n = 5$ for corn oil, and n = *6* for TC). The data were shown as mean ± SEM. Statistically significant pairwise comparisons were calculated using Ordinary one-way ANOVA tests; *p* values were determined by comparing to corn oil or And.

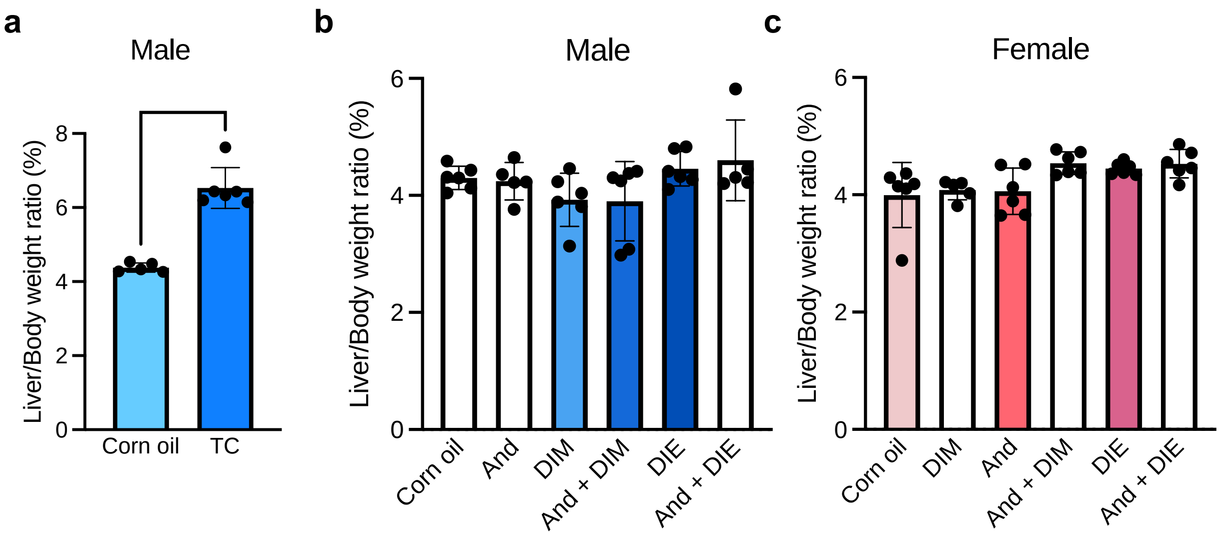

Supplementary Fig. 8 | Liver-to-body weight ratios in control and treatment groups of both sexes. a, liver-to-body weight ratio is significantly increased in WT male mice treated with TC. **b,c,** There is no difference in the liver-to-body weight ratio in diindole treatment groups compared to control groups. ($n = 6$ mice). Data are shown as means ± SEM.***$p < 0.001$ (two-tailed unpaired Student's t-test).

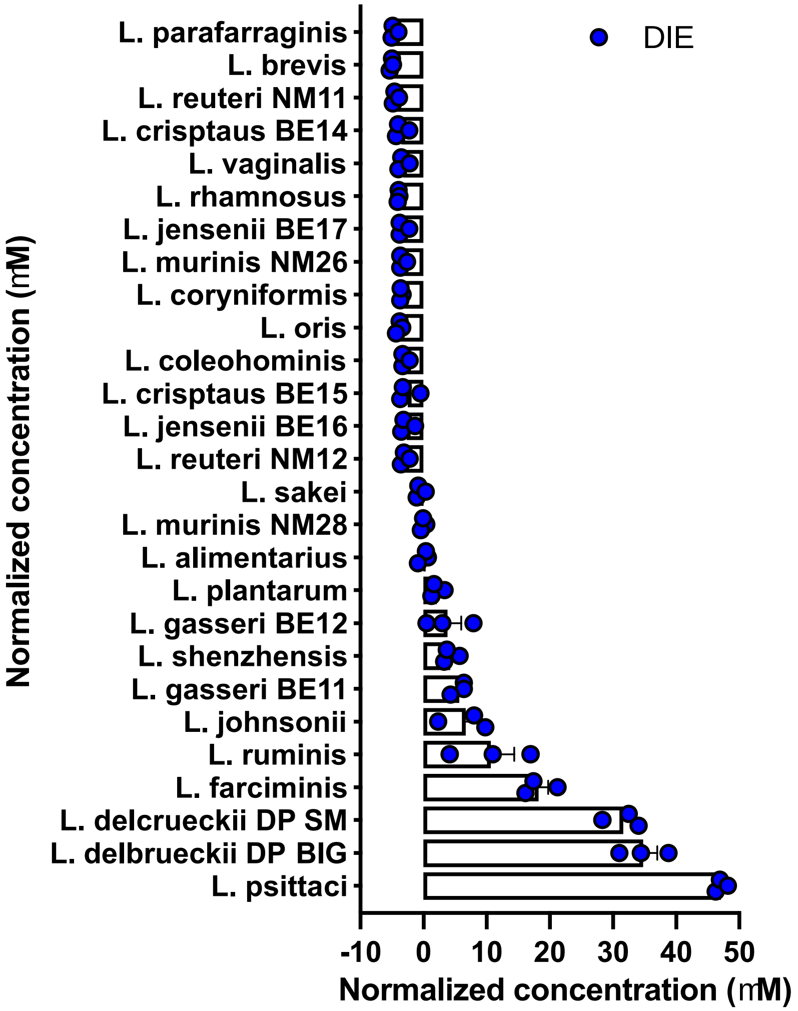

Supplementary Fig. 9 | Evaluation of DIE producing ability across a 27-strain Lactobacilli collection. Bacteria were incubated overnight with indole (500 μM) in MRS broth, and the negative control without indole (values are means ± SD based on triplicates, n = 3 independent experiments). Values were normalized to bacterial cell number by OD_{600} and corrected by subtraction of the negative control value.

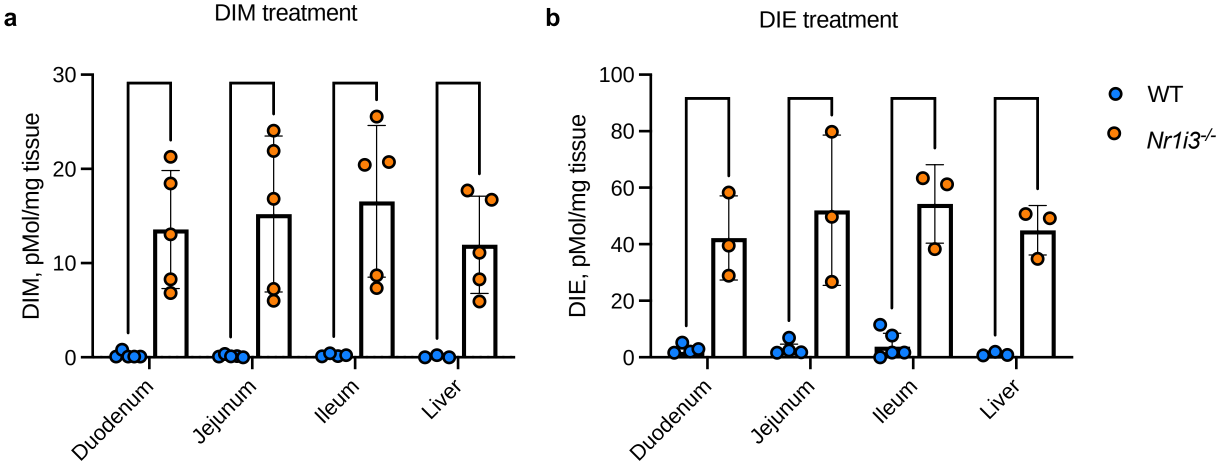

Supplementary Fig. 10 | Eleluation of diindole levels in the tissues of WT and *Nr1i3*$^{-/-}$ male mice treated with diindoles. Levels of DIM (**a**) and DIE (**b**) increased dramatically in all knockout animal tissues examined. The data were shown as mean ± SD (n = 5 mice for DIM and DIE in WT, and DIM in *Nr1i3*$^{-/-}$ mice, n = 3 mice for DIE in *Nr1i3*$^{-/-}$ mice). Statistically significant pairwise comparisons were calculated using Ordinary one-way ANOVA tests; p values were determined by comparing to WT control. ***p <0.001.

Supplementary Table 1 | ^{1}H and ^{13}C NMR data of GM-1, GM-2, and GM-7 in methanol-d_4 (δ in ppm, J in Hz).

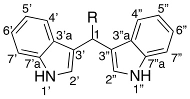

GM-1, R=H
GM-2, R=$\overset{2}{C}H_3$
GM-7, R=$\overset{2}{C}H(\overset{3}{C}H_3)_2$

C/H	GM-1		GM-2		GM-7		DIM	
	δ_H (J in Hz)	δc	δ_H (J in Hz)	δc	δ_H (J in Hz)	δc	δ_H (J in Hz)	δc
1	4.18, s	20.7, CH_2	4.69, q (7.0)	29.6, CH	4.55, q (7.0)	33.1, CH	4.18, s	22.2, CH_2
2			1.77, d (7.0)	22.5, CH_3	1.60, m	27.1, CH		
3					0.98 , d (7.0)	23.3, CH_3		
2' & 2"	6.94, s	122.1, CH	6.94, s	122.4, CH	6.87, s	122.7, CH	6.94, s	122.1, CH
3' &3"		114.6, C		106.4, C		106.4, C		116.1, C
3'a &3"a		127.5, C		128.3, C		128.4, C		128.9, C
4' &4"	7.50, d (7.8)	118.3, CH	7.42, d (7.9)	120.2, CH	7.49, d (8.0)	120.2, CH	7.50, d (7.8)	119.8, CH
5' & 5"	6.95, td (7.8, 8.1)	117.8, CH	6.86, t (7.9)	119.1, CH	6.88, dd (8.0, 2.0)	119.1, CH	6.95, td (7.8, 8.1)	119.3, CH
6' & 6"	7.03, t (7.8)	120.6, C	7.01, t (7.4)	122.0, C	7.00, dd (8.0, 2.0)	122.7, C	7.05, t (7.8)	123.5, C
7' & 7"	7.31, d (8.1)	110.7, CH	7.29,d (7.9)	112.1, CH	7.28, d (8.0)	112.1, CH	7.31, d (8.1)	112.1, CH
7'a & 7"a		138.3, C		138.5, C		138.4, C		138.3, C

Supplementary Table 2 | HDX summary table.

Data Set	Apo hCAR	Ligand DIE	Ligand CITCO
HDX reaction details	10 mM Phosphate buffer in D_2O, pD 7.00, 25°C		
HDX time course (min)	1, 10 and 30		
# of Peptides	136		
Sequence Coverage	94.1 %		
Average peptide length/ Redundancy	9.94/5.49		
Replicates (biological or technical)	3 technical replicates		
Repeatability	0.168 (average standard deviation)	0.161 (average standard deviation)	0.161 (average standard deviation)
Significant difference in HDX (delta HDX > x D)	0.492 (99% CI)		

Supplementary Table 3 | Primers for gene expression analysis.

Gene	Primer-Forward	Primer-Reverse
cyp2b10	TGCTGTCGTTGAGCCAACC	CCACTAAACATTGGGCTTCCT
cyp3a11	CTCTGGGTCTGTGACAGCAA	CAGCTTGGTGCTCCTCTACC
sult2a1	ACAGCTCTTTCCAAGCCATGA	CAGTCCCCAATTGTGCCTTT
36b4	AGATGCAGCAGATCCGCAT	GTTCTTGCCCATCAGCACC
cyp2b6	AGACGCCTTCAATCCTGACC	CCTTCACCAAGACAAATCCGC
cyp3a4	GTGGGGCTTTTATGATGGTCA	GCCTCAGATTTCTCACCAACACA
sult2a1	AGCGATCACCCTGGGT	TGGGAGGAGAATAAAC
gadph	CCCATCACCATCTTCCAGGAG	GTTGTCATGGATGACCTTGGC
