# Supplementary material for: Diindoles produced from commensal microbiota metabolites function as endogenous CAR/Nr1i3 ligands
Source: Nat Commun. 2024 Mar 22;15:2563. doi: 10.1038/s41467-024-46559-3 (PMC10960024; doi:10.1038/s41467-024-46559-3)
Supplement: Supplementary file 2 — Reporting Summary [file 41467_2024_46559_MOESM2_ESM.pdf]

nature portfolio

Corresponding author(s): Henry Krause

Last updated by author(s): Feb 25, 2024

Reporting Summary

Nature Portfolio wishes to improve the reproducibility of the work that we publish. This form provides structure for consistency and transparency in reporting. For further information on Nature Portfolio policies, see our Editorial Policies and the Editorial Policy Checklist.

Statistics

For all statistical analyses, confirm that the following items are present in the figure legend, table legend, main text, or Methods section.

n/a	Confirmed	
☐	☒	The exact sample size (*n*) for each experimental group/condition, given as a discrete number and unit of measurement
☐	☒	A statement on whether measurements were taken from distinct samples or whether the same sample was measured repeatedly
☐	☒	The statistical test(s) used AND whether they are one- or two-sided<br>*Only common tests should be described solely by name; describe more complex techniques in the Methods section.*
☒	☐	A description of all covariates tested
☒	☐	A description of any assumptions or corrections, such as tests of normality and adjustment for multiple comparisons
☐	☒	A full description of the statistical parameters including central tendency (e.g. means) or other basic estimates (e.g. regression coefficient) AND variation (e.g. standard deviation) or associated estimates of uncertainty (e.g. confidence intervals)
☒	☐	For null hypothesis testing, the test statistic (e.g. *F*, *t*, *r*) with confidence intervals, effect sizes, degrees of freedom and *P* value noted<br>*Give P values as exact values whenever suitable.*
☒	☐	For Bayesian analysis, information on the choice of priors and Markov chain Monte Carlo settings
☒	☐	For hierarchical and complex designs, identification of the appropriate level for tests and full reporting of outcomes
☒	☐	Estimates of effect sizes (e.g. Cohen's *d*, Pearson's *r*), indicating how they were calculated

Our web collection on statistics for biologists contains articles on many of the points above.

Software and code

Policy information about availability of computer code

Data collection	Thermo Fisher Xcalibur was used to operate LCMS system and collect the LCMS data.<br>The 1H and 13C NMR spectra were recorded on a 700 MHz Agilent DD2 NMR Spectrometer.<br>The TR-FRET emission was measured with a Tecan Infinite F200 Fluorescence Microplate Reader.<br>Luminescence was measured with a Tecan Infinite F200 Fluorescence Microplate Reader or Perkin Elmer, X5 2030 Multilabel Reader.<br>QuantStudio 12K Flex Real Time PCR System was used for thermal shift assay.<br>MicroCal Auto-iTC200 instrument was used for isothermal titration calorimetry.<br>Waters HDX system (M-class ACQUITY UPLC and Water Cyclic IMS Mass Spectrometer) was used for HDX experiment.<br>RT-PCR assay was performed on an ABI StepOnePlus Real-Time PCR System (Applied Biosystems).<br>The subcellular localization of hCAR and Nuclei were visualized by using a Nikon Eclipse Ti-E inverted fluorescence microscope (Nikon, Edgewood, NY)

Data analysis	The code used in the pulldown analysis is available from https://github.com/huiUofT/humanCAR. Specifically, raw MS files were converted to open format (.mzXML). Chemical features were detected with XCMS package at 2.5 ppm mass accuracy. Features were matched across different samples with a 0.5 min retention time tolerance window. Putative metabolite features were selected by calculating the fold change of features from treatments relative to controls.<br>NMR spectra were analyzed with MestRenova.<br>Integrated heat effects of ITC were analysed using Microcal Origin 7 software (Malvern Instruments).<br>ProteinLynx Global server (PLGS) and DynamX software was used for peptide identification and deuterium uptake analysis, respectively.<br>PyMOL 2.5 was used for structural visualization.<br>All statistical analyses were performed using GraphPad PRISM 9.

For manuscripts utilizing custom algorithms or software that are central to the research but not yet described in published literature, software must be made available to editors and reviewers. We strongly encourage code deposition in a community repository (e.g. GitHub). See the Nature Portfolio guidelines for submitting code & software for further information.

Data

Policy information about availability of data

All manuscripts must include a data availability statement. This statement should provide the following information, where applicable:

- Accession codes, unique identifiers, or web links for publicly available datasets
- A description of any restrictions on data availability
- For clinical datasets or third party data, please ensure that the statement adheres to our policy

Metabolomic data generated in this study have been deposited in MassIVE under accession code ID: MSV000091840 (https://doi.org/doi:10.25345/C5NV99M7M).
Protein structure used in this study has been deposited in PDB under code 1XVP (https://doi.org/10.2210/pdb1XVP/pdb).

Research involving human participants, their data, or biological material

Policy information about studies with human participants or human data. See also policy information about sex, gender (identity/presentation), and sexual orientation and race, ethnicity and racism.

Reporting on sex and gender	Not Applicable
Reporting on race, ethnicity, or other socially relevant groupings	Not Applicable
Population characteristics	Not Applicable
Recruitment	Not Applicable
Ethics oversight	Not Applicable

Note that full information on the approval of the study protocol must also be provided in the manuscript.

Field-specific reporting

Please select the one below that is the best fit for your research. If you are not sure, read the appropriate sections before making your selection.

[x] Life sciences [ ] Behavioural & social sciences [ ] Ecological, evolutionary & environmental sciences

For a reference copy of the document with all sections, see nature.com/documents/nr-reporting-summary-flat.pdf

Life sciences study design

All studies must disclose on these points even when the disclosure is negative.

Sample size	For mouse experiments, sample size was determined based on previous studies using WT and NR1i3-/- mice in CAR studies (PMID: 30620001, 33828301, 25661872,32875282) 3-6 mice each group were used in individual animal studies.
Data exclusions	No data were excluded.
Replication	At least three independent experiments were performed in pulldown, TSA, and luciferase reporter assays. Two independent experiments were performed in ITC, TR-FRET, and subcellular localization assay. All attempts were shown the same tendency. For mouse experiments, all the biological replicates to support reproducibility are described in each figure legend. All attempts at replication were successful.
Randomization	All animals with same genotype were randomly divided into control or experimental groups. Primary human hepatocytes CAR subcellular localization assay were randomly allocated into experimental groups.
Blinding	Images were unbiasedly collected and the analysis was performed by an investigator blinded to the samples. Investigators were not blinded to the sample groups in mouse experiment because the drug administration was repeated 3 days to complete the experiment, and the analysis was based on the genotypes.

Reporting for specific materials, systems and methods

We require information from authors about some types of materials, experimental systems and methods used in many studies. Here, indicate whether each material, system or method listed is relevant to your study. If you are not sure if a list item applies to your research, read the appropriate section before selecting a response.

Materials & experimental systems

n/a	Involved in the study
☒	☐ Antibodies
☐	☒ Eukaryotic cell lines
☒	☐ Palaeontology and archaeology
☐	☒ Animals and other organisms
☒	☐ Clinical data
☒	☐ Dual use research of concern
☒	☐ Plants

Methods

n/a	Involved in the study
☒	☐ ChIP-seq
☒	☐ Flow cytometry
☒	☐ MRI-based neuroimaging

Eukaryotic cell lines

Policy information about cell lines and Sex and Gender in Research

Cell line source(s)	HEK293T cell line (ATCC, CRL-3216); HepG2 (ATCC, HB-8065), HG5LN cell line was established by integration of a GAL4-responsive luciferase reporter gene (GAL4RE5-bGlob-Luc-SV-Neo) in HeLa cells; H4IIE-luc cell line was established by integration of a luciferase reporter gene under control of dioxin-responsive enhancers in rat hepatoma cell line.<br>Freshly isolated human primary hepatocytes (HPHs) with over 90% viability were obtained from BioIVT (Baltimore, MD). HL203 54yro Female Caucasian, HL204 61yro Female Caucasian.
Authentication	The cell lines used in this study were purchased or established by the authors. The authentication are not needed.
Mycoplasma contamination	All cell line were confirmed no mycoplasma contamination.
Commonly misidentified lines<br>(See ICLAC register)	No commonly misidentified cell lines were used in this study.

Animals and other research organisms

Policy information about studies involving animals; ARRIVE guidelines recommended for reporting animal research, and Sex and Gender in Research

Laboratory animals	12–15 week old C57BL/6J mice were obtained from Jackson Laboratories and were housed on a standard 12 hours light/dark cycle. Nr1i3-/- mice with C57BL/6J background were obtained from David Moore’s lab (PMID: 11057673) at Baylor College of Medicine, and used at 13–26 weeks of age.
Wild animals	This study did not involve wild animals.
Reporting on sex	Both wild-type male and female mice and Nr1i3-/- male mice were used in this study due to the different phenotypes in male and female mice (PMID: 34273530, 38149074).
Field-collected samples	No field collected samples were used in this study.
Ethics oversight	All experiments performed as directed by the Institutional Animal Care and Use Committee at the University of Illinois, Urbana-Champaign (UIUC, IACUC protocol: 22183).

Note that full information on the approval of the study protocol must also be provided in the manuscript.
